# When neighbors control metabolite fate: consequences for plant pathogen virulence and biocontrol outcomes

**DOI:** 10.1128/aem.00149-26

**Published:** 2026-04-21

**Authors:** Carlos N. Lozano-Andrade, Joana I. Pires Queiroz, Lars Jelsbak

**Affiliations:** 1 Microbiome Interactions and Engineering Group, Department of Biotechnology and Biomedicine, Technical University of Denmark 5205, Kgs. Lyngby, Denmark; Michigan State University, East Lansing, Michigan, USA

**Keywords:** antivirulence, biocontrol, plant pathogens, secondary metabolites, biotransformation, cyclic lipopeptide, microbial interaction

## Abstract

Microbial interactions can profoundly influence the activity of bioactive metabolites in plant-associated communities. De Rop et al. show that community members enzymatically degrade cyclic lipopeptides (CLiPs), metabolites that act both as virulence factors in plant pathogens and antimicrobial agents in biological control (J. De Rop, D. Prasad, N. Geudens, L. Zhou, et al., Appl Environ Microbiol 92:e01524-25, 2026, https://doi.org/10.1128/aem.01524-25). By neutralizing these molecules, microbes determine whether CLiP-mediated activities persist or collapse, revealing that metabolite function depends on community-mediated turnover and highlighting an underappreciated ecological layer that shapes plant disease and biocontrol outcomes.

## COMMENTARY

**Fig 1:**
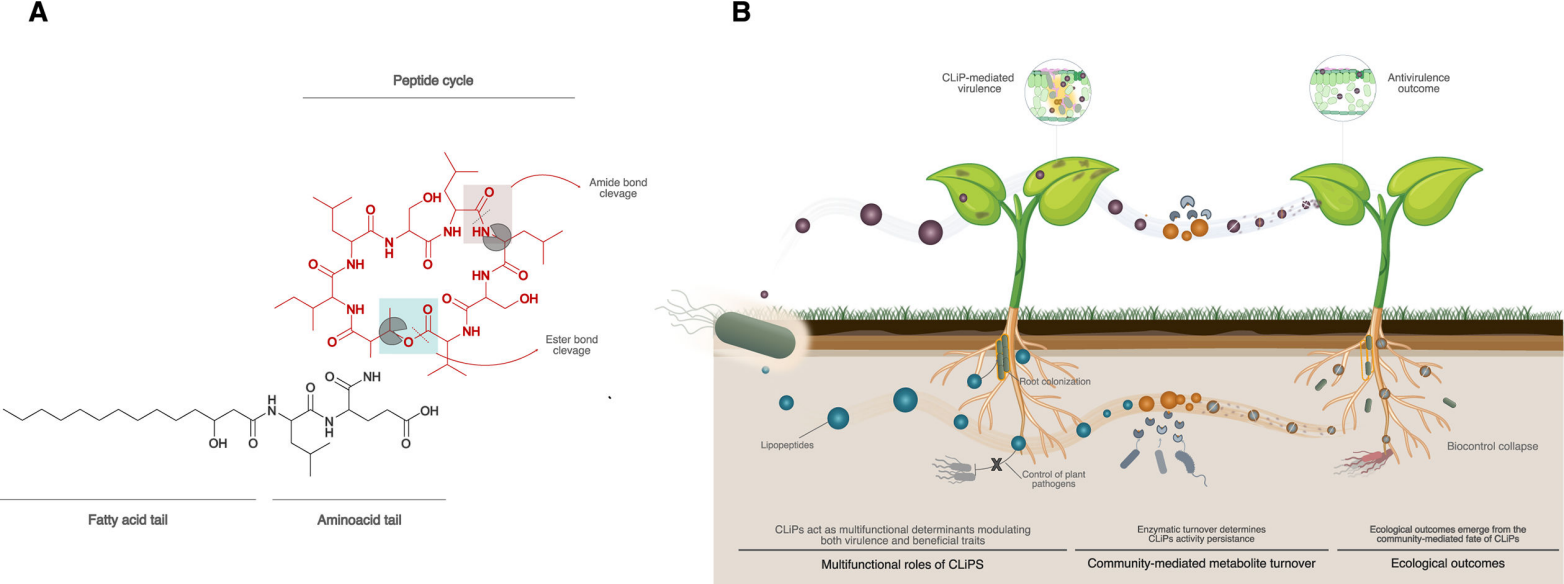
Community-mediated turnover shapes the ecological roles of cyclic lipopeptides (CLiPs). (**A**) Structural organization of CLiPs, comprising a fatty acid tail linked to a cyclic oligopeptide. Enzymatic cleavage can occur at distinct sites, including at ester or amide bonds in the macrocyclic ring, thereby altering CLiP structure and activity. (**B**) Conceptual model illustrating how community-mediated turnover modulates CLiP function in plant-associated environments. CLiPs act as multifunctional determinants mediating both virulence and beneficial traits. In pathogenic contexts, CLiP persistence enables host damage, whereas enzymatic degradation reduces virulence. In beneficial contexts, CLiPs promote root colonization and pathogen suppression, but turnover compromises metabolite persistence and can lead to biocontrol collapse. Thus, identical degradation processes yield distinct ecological outcomes depending on context, underscoring that metabolite persistence, and not production alone, is a key determinant of CLiP function.

Cyclic lipopeptides (CLiPs) are a structurally diverse class of small bioactive secondary metabolites produced by many plant-associated bacteria, including beneficial rhizobacteria such as *Pseudomonas* and *Bacillus*, as well as several plant pathogens. These amphiphilic molecules consist of an oligopeptide that is cyclized through an ester bond and linked to a fatty acid tail (1–4) (Fig. 1A). Across plant-associated bacteria, CLiPs function as multifunctional “interaction tools” (Fig. 1B). In plant-beneficial species, CLiPs display broad antimicrobial activity and can stimulate plant immune responses, contributing to induced systemic resistance and their application in biological control of phytopathogens (5, 6). In contrast, in plant-pathogenic bacteria, CLiPs frequently function as virulence factors or toxins that facilitate host colonization through membrane disruption, tissue damage, or suppression of plant defenses (7–9). Beyond their direct effects on plants, CLiPs also influence microbial interactions in the rhizosphere by mediating competition and enabling traits such as motility, biofilm formation, and root colonization (4, 10, 11).

Despite their ecological importance, most research has examined CLiPs in the context of biosynthesis, regulation, and phenotypic outcomes in single-species systems where these metabolites are implicitly treated as chemically stable end products. However, soil and rhizosphere microbiomes are dynamic environments populated by metabolically active organisms capable of transforming exogenous compounds and metabolites secreted by other microbes and plants. The demonstration by De Rop et al. (12) that neighboring microbes can degrade CLiPs suggests that CLiP-mediated virulence as well as biocontrol antagonism should be viewed not only as consequences of metabolite production alone, but as outcomes of community-level metabolite turnover that may vary with environmental conditions and microbiome composition (Fig. 1B).

## THE CHEMICAL FATE OF CLiPS IN MICROBIAL INTERACTIONS

The study by De Rop et al. is indeed a clear demonstration that CLiPs can be actively remodeled during interspecies interactions, with direct ecological consequences (12). The authors investigate chemical interactions between two rice sheath pathogens: the bacterium *Pseudomonas fuscovaginae*, the causal agent of sheath brown rot, and the fungus *Rhizoctonia solani* AG 1-IA, responsible for sheath blight. Their central aim was to define the “chemical fate” of two bacterial CLiPs during interactions with the fungal competitor: syringotoxin (a mycin-type CLiP) and fuscopeptin (a peptin-type CLiP). Both CLiPs display potent antifungal activity against *R. solani* while simultaneously causing phytotoxic effects. Using cell-free supernatants combined with targeted LC/UPLC-MS/MS analyses, they demonstrate that the fungus enzymatically neutralizes both CLiPs through extracellular activities, abolishing their antifungal activity and phytotoxic effects. Strikingly, the two molecules are inactivated via distinct chemical routes consistent with different enzymatic mechanisms. Syringotoxin accumulates a +18 Da product indicative of ester or lactone hydrolysis and ring opening, whereas fuscopeptin undergoes site-specific cleavage within the peptide backbone at a glycine–alanine bond, consistent with proteolytic attack. Importantly, CLiP biotransformation is environmentally tuned. Degradation activity increases with temperature and is enhanced during fungal growth at 28°C, a regime associated with the ecological dominance of *R. solani* over *P. fuscovaginae*. In contrast, sheath brown rot caused by *P. fuscovaginae* is more prevalent under cooler, high-altitude conditions. The temperature dependence of the CLiP degradation, together with its conservation across multiple AG 1-IA isolates, suggests that CLiP neutralization is not incidental but represents an evolved ecological strategy shaping competitive interactions between these two pathogens. De Rop et al. therefore propose a model of temperature-dependent antagonism underlying the apparent mutual exclusivity of these pathogens: at 18°C, elevated CLiP production enhances bacterial virulence and suppresses fungal growth, whereas at 28°C, reduced CLiP production combined with enhanced fungal enzymatic degradation shifts the competitive balance in favor of *R. solani* AG 1-IA. Together, the work reframes CLiPs not simply as bioactive metabolites but as transient chemical currencies whose ecological impact depends on the environmental context and the metabolic countermeasures of competing microbes.

## VIRULENCE AS A COMMUNITY-MODULATED TRAIT

From an applied perspective, the study by De Rop et al. is a striking example of how microbial interactions can modulate virulence traits by effectively “disarming” a pathogen rather than eliminating it or inhibiting its growth (Fig. 1B). Importantly, this phenomenon is not isolated but reflects an emerging theme in host-associated microbiomes, where neighboring microorganisms interfere with key virulence factors of invading pathogens. A compelling parallel example is found in the mushroom pathogen *Pseudomonas tolaasii*, the causative agent of brown blotch disease in *Agaricus bisporus*, which causes substantial losses in mushroom production. During infection, *P. tolaasii* relies on two cyclic lipopeptides: the pore-forming toxin tolaasin and the biosurfactant pseudodesmin, the latter enabling swarming motility across surfaces. Remarkably, mushroom-associated “helper bacteria” of the genus *Mycetocola* protect their fungal host by enzymatically linearizing and inactivating both pathogen-produced CLiP molecules through hydrolysis of their macrocyclic ring, thereby suppressing toxin activity as well as motility-driven dissemination of the pathogen (13).

Together, these studies define a broader conceptual framework in which microorganisms, and the enzymes they deploy, can control pathogens by neutralizing the chemical determinants of virulence. Such antivirulence strategies aim to suppress disease by disabling toxins, surfactants, signaling molecules, and other secreted effectors rather than inhibiting pathogen growth directly. This approach holds particular promise for sustainable agriculture, as it may preserve beneficial microbiota while reducing selective pressure for resistance that is typically associated with antibiotics and other agrochemicals that target pathogen growth.

A key next step will be to determine how widespread such metabolite inactivation processes are in natural microbiomes and whether they represent rare specialized interactions or a more general ecological principle. Ultimately, exploiting this hidden layer of microbial interaction may shift crop protection strategies from killing pathogens toward steering microbiomes into antivirulence states that render pathogens harmless, including through the deployment of microorganisms or enzymes capable of disarming pathogen-derived virulence factors.

## BIOCONTROL AS A COMMUNITY-MODULATED TRAIT

While some CLiP serve as potent virulence factors in phytopathogens, others function as antimicrobial metabolites capable of suppressing fungal pathogens across diverse environments. Their strong antifungal activity has made them attractive tools for sustainable crop protection, and CLiP-producing *Pseudomonas* strains are incorporated as active ingredients in commercial biocontrol products (14). However, the recognition that microbial competitors can enzymatically inactivate virulence-associated CLiPs, as shown by De Rop et al., raises an important parallel question for beneficial microbes: what is the ecological fate of CLiPs deployed as biocontrol agents?

Biocontrol research has often implicitly assumed that once produced, CLiPs remain chemically intact and exert predictable antimicrobial effects in soil and rhizosphere environments. Yet the findings of De Rop et al. suggest that these antimicrobial metabolites may instead represent transient ecological agents whose activity depends on the surrounding microbial community, which may either attenuate or prolong metabolite activity.

Research into CLiP stability, persistence, and degradation under natural soil and rhizosphere conditions remains limited. However, emerging evidence shows that *Pseudomonas*-produced CLiPs can be degraded by soil and rhizosphere microorganisms. For instance, when added exogenously to soil, viscosinamide (and other *Pseudomonas* CLiPs) is rapidly degraded, with a half-life of approximately 5 days. This degradation activity is abolished by soil sterilization, indicating that indigenous soil microorganisms have the capacity to degrade viscosinamide, although the specific responsible taxa were not identified (15).

More recently, we found that orfamide, a CLiP produced by the well-characterized plant-beneficial *P. protegens* species, is degraded *in vitro* through the concerted actions of a four-species synthetic community of soil bacteria (16). Orfamide degradation proceeds as a two-step process: one community member (*Rhodococcus* spp.) first linearizes the cyclic orfamide molecule by hydrolyzing the ester bond that closes the macrocyclic ring. This linearized form of orfamide, but not the cyclic molecule, then becomes accessible for another community member (*Stenotrophomonas* spp.), which degrades the peptide backbone through successive cleavage of amide bonds.

These observations also suggest that CLiP degradation can substantially expand the chemical space emerging from microbial interactions, generating diverse transformation products with potentially novel ecological functions. Rather than simply abolishing activity, enzymatic modifications may produce metabolites with altered or entirely new bioactivities. For example, degradation of the *Pseudomonas* lipopeptide syringafactin by *Paenibacillus* generates compounds that are toxic to amoebal predators. This case illustrates a dual microbial interaction in which one species produces a bioactive lipopeptide, while a partner species enzymatically reshapes it, effectively repurposing the metabolite into new functional molecules that influence predator–prey dynamics and microbial competition (17).

Recent work further proposes that CLiP degradation may also serve metabolic functions within microbial communities. Rigolet et al. showed that *Streptomyces* spp. degrade lipopeptides produced by both *Bacillus* and *Pseudomonas* and suggested that this process may represent a foraging strategy enabling access to nutrients derived from competing microorganisms (18). Collectively, these findings indicate that CLiP degradation represents not merely metabolic inactivation but rather a process of chemical diversification, generating secondary waves of molecules and transformation products that may influence microbial physiology and reshape community dynamics.

Together, these examples clearly illustrate that CLiPs are subject to active, community-mediated turnover in soil environments. If CLiP inactivation is widespread in soil communities, then the variable performance often observed in biocontrol applications may partly reflect a mismatch between production by the introduced biocontrol strain and the persistence of active CLiP molecules within the resident soil microbiome (Fig. 1B). A testable prediction is that disease suppression should then correlate with (i) the abundance or expression of candidate CLiP-hydrolase genes and/or (ii) measurable extracellular hydrolytic activity against representative CLiPs in a given soil. This framework offers a mechanistic explanation for the long-recognized context dependency of biocontrol performance across soils. Incorporating this “chemical fate” layer into screening could therefore shift biocontrol development from selecting the highest-producing strain *in vitro* to identifying and selecting strains (as well as deployment contexts) in which bioactive CLiPs persist and remain active for long enough to influence pathogen growth or host responses.

## TOWARD A MECHANISTIC UNDERSTANDING OF CLiP BIOTRANSFORMATIONS

The finding by De Rop et al. that fungal species can chemically modify CLiP molecules adds a new dimension to the growing body of evidence for CLiP biotransformations. An emerging pattern is that these modifications frequently rely on a limited set of recurrent biochemical reactions, notably cleavage of macrocyclic ester bonds and, in some cases, hydrolysis of amide bonds (Fig. 1A). Strikingly, such transformations occur across diverse ecological niches—including the rhizosphere, phyllosphere, and mushroom microbiomes—and across phylogenetically distant lineages spanning Actinobacteria, Proteobacteria and, as now demonstrated by De Rop et al., fungi.

Identifying the enzymes responsible for CLiP modification, therefore, represents a critical next step. Without mechanistic insight, metabolite turnover remains a descriptive observation rather than a predictable ecological process. To date, only a few such enzymes have been characterized, but recent work has begun to uncover the specific enzymatic mechanisms underlying lipopeptide transformation. For example, in the mushroom–pathogen–helper bacterium system described above, activity-guided proteome fractionation combined with heterologous expression identified a hydrolase responsible for cleavage of the macrocyclic ester of the tolaasin toxin (13). Similarly, the hydrolase SfhA cleaves the ester bond of surfactin, a cyclic lipopeptide produced by *Bacillus subtilis* (19). SfhA was identified in *Streptomyces* Mg1 through secretome proteomics, enabling purification of an enzyme capable of hydrolyzing surfactin *in vitro*. Finally, returning to the cooperative interaction in which *Paenibacillus* modifies the *Pseudomonas*-derived lipopeptide syringafactin to generate predator-toxic products, Zhang et al. identified two lipopeptide-induced DL-peptidases, Lip3 and Lip7, that selectively cleave DL-amide bonds within syringafactin. By exposing *Paenibacillus* to syringafactin, the authors pinpointed peptidase genes specifically upregulated in response to the metabolite, followed by heterologous expression and functional screening of candidate enzymes. Only two proteins displayed lipopeptide-cleaving activity, revealing enzymes that remodel the molecule and thereby transform its biological function (20).

Future discovery and characterization of CLiP-modifying enzymes will help reveal the specificity, regulation, and evolutionary origins of metabolite neutralization and may ultimately allow competitive interactions to be inferred directly from genomic potential. Growing evidence, including the cross-kingdom CLiP modifications described by De Rop et al., indicates that such processes are widespread features of microbial communities. Uncovering the enzymatic logic of metabolite transformation will therefore be essential for understanding and, ultimately, controlling the chemical interactions that shape microbial ecosystems.

Rather than viewing bioactive metabolites as static weapons, we suggest that they are better understood as dynamic ecological currencies whose activity depends on community context and enzymatic turnover (Fig. 1B). This shift may open new avenues for the rational design of antivirulence and biocontrol strategies for sustainable crop protection, shifting attention from microbial metabolite production alone toward understanding and managing the ecological and enzymatic processes that govern how bioactive molecules persist, transform, and function *in situ* within plant-associated microbial communities.
